# Enhanced oral glucose tolerance test for early detection of insulin resistance and metabolic complications in children with obesity

**DOI:** 10.1016/j.ajpc.2025.101016

**Published:** 2025-06-04

**Authors:** Urh Groselj, Jan Kafol, Jaka Sikonja, Matej Mlinaric, Robert Sket, Ziga Iztok Remec, Jernej Kovac, Ana Drole Torkar, Jasna Suput Omladic, Barbka Repic Lampret, Tadej Battelino, Primoz Kotnik

**Affiliations:** a Department of Endocrinology, Diabetes, and Metabolic Diseases, University Children's Hospital, University Medical Centre Ljubljana, Ljubljana, Slovenia; b Faculty of Medicine, University of Ljubljana, Ljubljana, Slovenia; c Department of Vascular Diseases, Division of Internal Medicine, University Medical Centre Ljubljana, Ljubljana, Slovenia; d Department of Endocrinology, Diabetes and Metabolic Diseases, Division of Internal Medicine, University Medical Centre Ljubljana, Ljubljana, Slovenia; e Clinical Institute of Special Laboratory Diagnostics, University Children's Hospital, University Medical Centre Ljubljana, Ljubljana, Slovenia

**Keywords:** Insulin resistance, Oral glucose tolerance test, OGTT, Metabolic complications, Screening, Children, Adolescents, Obesity

## Abstract

**Background and aims:**

Early detection of insulin resistance (IR) and obesity-related complications is crucial for preventing type 2 diabetes. This study aimed to identify dynamic metabolic biomarkers for more precise early detection of IR and metabolic abnormalities.

**Methods:**

This cross-sectional cohort study evaluated IR and metabolic biomarkers in 403 children with obesity (median age 13.18 years, 51.3 % female, 98.5 % with obesity) using an enhanced oral glucose tolerance test (eOGTT). IR was assessed via four indices, with the Matsuda Insulin Sensitivity Index (ISI-M) used as the primary measure. Participants were stratified into quartiles based on ISI-M.

**Results:**

Participants with the highest IR (Q1) were older (*p* = 0.002), had a higher body mass index, were in a more advanced pubertal stage (*p* < 0.001), and had significantly elevated glucose and insulin levels (*p* < 0.001 for both) compared to the most insulin sensitive (Q4), with significant differences observed across all quartiles (*p* < 0.050 for all). Insulin at 120 min demonstrated excellent diagnostic accuracy for IR (AUC=0.958). Triglyceride levels in Q1 showed minimal decline during the eOGTT, while greater declines were observed with increasing insulin sensitivity (*p* = 0.002 across quartiles), suggesting that a lack of decline in triglycerides may help identify IR. High-sensitivity C-reactive protein levels increased with IR (*p* = 0.024). Baseline beta-hydroxybutyrate levels were highest in the Q4 and showed the greatest absolute decrease during the eOGTT, compared to Q1 (*p* < 0.001 for both).

**Conclusions:**

We validated established IR markers in children with obesity, while demonstrating that eOGTT may offer improved characterization and earlier identification of those at risk for metabolic complications.

## 1 Introduction

Over the past decades, the prevalence of child overweight and obesity has rapidly increased, leading to psychological and physical health challenges and increasing the long-term risks for the development of cardiovascular disease, diabetes, certain cancers, and musculoskeletal disorders in adulthood[1,2].

Insulin resistance (IR), commonly linked with obesity, is a condition of impaired insulin-stimulated glucose uptake in muscle and adipose tissue, together with reduced insulin suppression of hepatic glucose production[3]. Fluctuations in insulin sensitivity are a normal part of the life cycle, with physiological IR occurring during puberty, pregnancy, and aging[[4], [5], [6]].

The development of IR in obesity is driven by dysfunctional adipose tissue, which releases free fatty acids (FFA), reactive oxygen species, and pro-inflammatory cytokines. Over time, this leads to low-grade systemic inflammation, further worsening IR[7]. Diabetes occurs when the accompanying increase in insulin secretion can no longer compensate for the level of IR[8]. Therefore, accurate and early detection of IR is essential to delay or prevent the onset of pre-diabetes and type 2 diabetes (T2D)[9].

The hyperinsulinemic-euglycemic clamp method is considered the gold standard for assessing insulin sensitivity. However, due to its complexity, its high cost, time-intensive nature, and labor demands, it is not suitable for epidemiological studies, large-scale clinical investigations, or routine clinical applications[10,11]. To overcome this limitation, various surrogate indexes of IR have been developed from loading tests, such as oral glucose tolerance test (OGTT) and mixed meal tolerance test, and from stable-state fasting plasma concentrations[12]. There is a growing need for reliable biomarkers for early identification of children and adolescents with IR that have a higher risk for the development of T2D and cardiometabolic complications, particularly with the ongoing obesity pandemic and emerging therapeutic options[13].

In this study, we performed an enhanced OGTT (eOGTT) to identify metabolic biomarkers that provide a more precise early determination of metabolic complications of obesity in a cohort of children and adolescents with overweight and obesity beyond the markers of IR detectable in the fasting steady state.

## 2 Materials and methods

### 2.1 Study design

This cross-sectional, single-center study aimed to identify biomarkers for early detection of IR and metabolic abnormalities in children and adolescents with overweight or obesity, using eOGTT. The study was approved by the Slovenian National Medical Ethics Committee (No. 29/02/13) and conducted in accordance with the Declaration of Helsinki. Written informed consent was obtained from the parents or caregivers of each participant.

### 2.2 Study participants

We screened for eligibility 1664 children and adolescents with overweight or obesity referred to the University Children’s Hospital Ljubljana (2010–2021) for: body mass index (BMI) >98th percentile for age and sex, suspected genetic or hormonal causes of obesity, and/or obesity-related medical complications (e.g., impaired glucose homeostasis, menstrual irregularities, hyperandrogenism, hyperlipidemia, hypertension, fatty liver, respiratory or sleep disorders, and orthopedic issues). Inclusion criteria included a body weight or BMI >85th percentile (or Z-score >1), age 2–20 years, and availability of anthropometric and laboratory data (**Supplementary Table 1**), with exclusion criteria detailed in **Supplementary Materials**. A total of 403 individuals met the inclusion and exclusion criteria and were included in the final analysis. To evaluate possible selection bias, we compared baseline anthropometric and fasting biochemical variables of the analytic cohort with those of the full registry (see **Supplementary Table 2**).

### 2.3 Study visit

Each participant was assessed by a pediatric endocrinologist who took a clinical history, performed a physical examination, and a pubertal stage assessment (**Supplementary Materials**). Anthropometric measurements (height, weight, waist, and hip circumference) were obtained using established methods, and British 1990 reference data with the LMS growth Excel add-in (Available at: http://www.healthforallchildren.com/shop-base/shop/software/lmsgrowth/) was used to calculate Z-scores[14,15]. Obesity was defined as BMI>95th percentile for age and sex[16]. Blood pressure and heart rate were measured in a seated position after rest using an automated sphygmomanometer.

### 2.4 Biochemical analysis and enhanced oral glucose tolerance test

Venous blood samples were collected in a fasting state at the University Medical Centre Ljubljana, both before their first meal and after undergoing the eOGTT. During the eOGTT, participants received 1.75 g of glucose per kilogram of body weight (up to 75 g) after the first blood draw (t0), with a second sample collected 120 min later (t120). Where necessary, samples were centrifuged within 30 min of collection. To minimize venipuncture burden, our institutional pediatric protocol mandates only two samples (fasting and 120 min); therefore no 30-minute blood draw was performed.

Glucose, liver enzymes (aspartate transaminase [AST], alanine transaminase [ALT], gamma-glutamyl transpeptidase [GGT]), total cholesterol (TC), cholesterol in high-density lipoprotein (HDL-C), directly measured cholesterol in low-density lipoprotein (LDL-C), and triglycerides (TAG) were measured in serum using the Abbott Alinity C Chemistry Analyzer (Abbott Laboratories, USA). Lipoprotein(a) [Lp(a)], apolipoprotein B (ApoB), apolipoprotein AI (ApoAI), and high-sensitivity C-reactive protein (hs-CRP) were determined from serum using immunonephelometry with the Siemens Nephelometer Analyzer Atellica Neph 630 (Siemens Healthineers, Ireland). FFA were determined using an enzymatic colorimetric method, beta-hydroxybutyrate (BHB) and l-carnitine were assessed with an enzymatic UV test on the Alinity C automated analyzer (Abbott Laboratories, USA). Insulin serum was measured using immunochemical methods with the Siemens Atellica analyzer (Siemens Healthineers, Ireland).

### 2.5 Estimation of insulin resistance

IR was estimated using four indices: Homeostasis Model Assessment for Insulin Resistance (HOMA-IR) [17], Matsuda Insulin Sensitivity Index (ISI-M) [18,19], Single Point Insulin Sensitivity Estimator (SPISE; calculated at t0 and t120) [20,21], and Quantitative Insulin Sensitivity Check Index (QUICKI)[22]. Detailed index calculations are provided in **Supplementary Materials**. ISI-M was the primary measure of whole-body insulin resistance (WBIR) since: several studies have demonstrated the high accuracy and superior diagnostic ability of ISI-M [19,[23], [24], [25]]; ISI-M also incorporates parameters during glucose loading in its calculation, unlike other indices of IR that are solely calculated from a fasting steady state; and a correlation analysis on our cohort demonstrated good correlation of ISI-M with other IR indices (**Supplementary Fig. 1**).

### 2.6 Statistical analysis

Data were analyzed using SPSS Statistics 26.0 (IBM), Excel 365 (Microsoft), and R 4.4.1 (R Foundation). Because the eligibility panel (fasting + 120-min glucose, insulin, full lipid profile, ApoAI, ApoB, liver enzymes) was an inclusion criterion, these variables were complete for all 403 participants; only ancillary biomarkers showed ≤ 4 % random missingness, so we analysed complete cases and did not impute. Outliers were included except for hs-CRP values >6 mg/L (*N* = 64), excluded to account for potential infection at sampling. The cohort was stratified into quartiles of WBIR, with Q1 indicating highest IR (lowest ISI-M index) and Q4 the lowest IR (highest ISI-M index). Numerical data were assessed for normality using Shapiro-Wilk, Kolmogorov-Smirnov, and graphical methods. Non-normally distributed data were reported as median (interquartile range [Q1–Q3]). Levene’s test assessed variance equality. Non-parametric tests (Mann-Whitney U, Kruskal-Wallis, Wilcoxon rank-sum) or parametric tests (T-test, ANOVA, Welch’s ANOVA for unequal variances) were applied based on distribution. Categorical data were analyzed using Chi-Squared Tests with Monte Carlo simulations for larger tables. Correlations were evaluated using Pearson’s (normal distribution) or Spearman’s methods (non-normal distribution). Receiver Operating Characteristic (ROC) curves assessed diagnostic performance of insulin and glucose metrics. Statistical significance for all tests was set at *p* ≤ 0.05, and for comparisons of multiple groups, post-hoc analyses (Dunn’s test for Kruskal-Wallis and Welch ANOVA, and Tukey’s test for ANOVA) were conducted for significant results. False discovery rates were controlled using the Benjamini-Hochberg correction.

## 3 Results

### 3.1 Baseline demographics and clinical characteristics

The study included 403 children and adolescents (51.3 % female), median age of 13.18 years (10.45–15.21). The majority (98.5 %) had obesity. Demographic and clinical characteristics by WBIR quartile groups are summarized in Table 1. No significant sex differences were observed across WBIR groups (*p* = 0.648). The most insulin-resistant participants (Q1) were older (*p* = 0.013) and had higher BMI Z-scores (*p* < 0.001), while there were no differences in height Z-scores (*p* = 0.672), implying that the increased BMI is attributable to increased weight. Waist-to-height ratio was also highest in Q1 (*p* = 0.001), whereas differences in waist-to-hip ratio were insignificant (*p* = 0.670).

**Table 1:** Baseline Demographics and Clinical Characteristics.

	Overall	Q1*	Q2	Q3	Q4	p-value†	p-value§
**Demographic characteristics**							
**Group size**	403	101	101	100	101		
**Females**	207 (51.3 %)	50 (49.5 %)	58 (57.4 %)	50 (50.0 %)	49 (48.5 %)	0.648^a^	1.000^b^
**Age, year**	13.18 (10.45– 15.21)	13.65 (11.73–15.71)	13.21 (11.19–14.75)	13.12 (10.65–15.01)	12.27 (8.59–15.04)	0.013^c^	0.002^d^
**Anthropometric characteristics**							
**Weight, kg**	78.5 (62.1–94.9)	90.3 (75.8–110.3)	79.7 (65.6–92.6)	76.4 (62.1–90.0)	65.6 (50.1–86.2)	<0.001^c^	<0.001^d^
**Weight Z-score**	2.85 (2.26–3.40)	3.24 (2.68–3.66)	2.81 (2.42–3.23)	2.74 (2.07–3.22)	2.67 (2.11–3.45)	<0.001^e^	<0.001^f^
**Height, cm**	162.0 (151.4– 171.2)	166.6 (156.5– 173.9)	161.4 (151.5– 171.2)	163.1 (153.0–169.9)	155.4 (139.8–168.5)	<0.001^c^	<0.001^f^
**Height Z-score**	1.02 (−0.05 to 1.90)	0.86 (−0.08 to 1.67)	1.02 (−0.11 to 1.83)	1.31 (0.24–2.08)	1.13 (0.09–1.93)	0.672^c^	0.628^f^
**BMI, kg/m^2^**	29.43 (26.47–33.17)	32.69 (28.74–36.55)	30.09 (27.42–33.02)	28.48 (26.08–31.46)	26.71 (24.70–30.28)	<0.001^c^	<0.001^d^
**BMI Z-score**	2.95 (2.55–3.35)	3.23 (2.85–3.51)	2.96 (2.74–3.25)	2.76 (2.49–3.20)	2.79 (2.24–3.37)	<0.001^g^	<0.001^d^
**Waist circumference, cm**	96 (88–105)	100.5 (92.3–113.8)	96 (89.8–104.5)	96 (88.5–102)	89 (80–97)	<0.001^c^	<0.001^f^
**Waist circumference Z-score**	3.15 (2.69–3.61)	3.35 (2.91–3.70)	3.15 (2.69–3.57)	3.12 (2.59–3.55)	3.18 (2.64–3.67)	0.366^c^	0.396^d^
**Hip circumference, cm**	104 (95–114)	113 (102–123)	104.5 (96–112.3)	103 (95–109)	100.5 (87.3–109)	<0.001^c^	<0.001^f^
**Waist-to-height ratio**	0.60 (0.56–0.65)	0.62 (0.58–0.69)	0.61 (0.55–0.66)	0.59 (0.55–0.63)	0.59 (0.55–0.62)	0.001^c^	<0.001^d^
**Waist-to-hip ratio**	0.94 (0.89–0.99)	0.95 (0.89–0.99)	0.95 (0.89–1.01)	0.94 (0.90–0.98)	0.93 (0.88–0.98)	0.670^c^	0.563^d^
**Systolic pressure, mmHg**	126 (117–135)	130 (124–138)	129 (117–134)	123 (117–135)	119 (112–131)	<0.001^e^	<0.001^f^
**Diastolic pressure, mmHg**	66 (59–72)	70 (65–75)	66 (60–72)	63 (58–70)	63 (57–69)	<0.001^e^	<0.001^d^
**Pubertal status**							
**Prepubertal**	68 (16.9 %)	8 (7.9 %)	14 (13.9 %)	16 (16.0 %	30 (29.7 %)	0.002^a^	<0.001^a^
**Midpubertal**	115 (28.5 %)	27 (26.7 %)	30 (29.7 %)	34 (34.0 %)	24 (23.8 %)		
**Late pubertal**	190 (47.1 %)	58 (57.4 %)	50 (49.5 %)	45 (45.0 %)	37 (36.6 %)		

Data are absolute frequency (proportion in %) and median (first quartile–third quartile).

**Legend:** Q – Quartile; BMI – Body mass index.

⁎ Q1 represents individuals with the highest insulin resistance;.

† p-value for comparisons between all four groups;.

§ p-value for comparisons between Q1 and Q4;.

a Monte Carlo Significance Test;.

b Chi-Squared Test;.

c Kruskal-Wallis Test;.

d Mann-Whitney Test;.

e ANOVA;.

f Independent Samples T-test;.

g Welch's ANOVA. Post-hoc comparisons for significant results were performed using Tukey's test for ANOVA or Dunn's test for Kruskal-Wallis and Welch’s ANOVA, with p-values adjusted using the Benjamini-Hochberg method (significance set at adjusted *p* < 0.05). Detailed results for significant comparisons are provided below (parameter name: adjusted p-value for Q2 vs. Q1, Q3 vs. Q1, Q4 vs. Q1, Q3 vs. Q2, Q4 vs. Q2, Q4 vs. Q3): Age: 0.205, 0.162, 0.003, 0.811, 0.116, 0.136; Weight: 0.003, <0.001, <0.001, 0.335, <0.001, 0.007; Weight Z-score: 0.050, 0.005, 0.005, 0.997, 0.997, 1.000; Height: 0.091, 0.119, <0.001, 0.822, 0.035, 0.028; BMI: 0.002, <0.001, <0.001, 0.039, <0.001, 0.032; BMI Z-score: 0.009, <0.001, <0.001, 0.094, 0.155, 0.728; Waist circumference: 0.038, 0.006, <0.001, 0.388, 0.003, 0.030; Hip circumference: 0.014, 0.001, <0.001, 0.363, 0.033, 0.189; Waist-to-height ratio: 0.113, 0.005, <0.001, 0.185, 0.079, 0.545; Systolic pressure: 0.007, <0.001, <0.001, 0.256, 0.002, 0.040; Diastolic pressure: 0.011, <0.001, <0.001, 0.099, 0.014, 0.386.

**Fig. 1:**
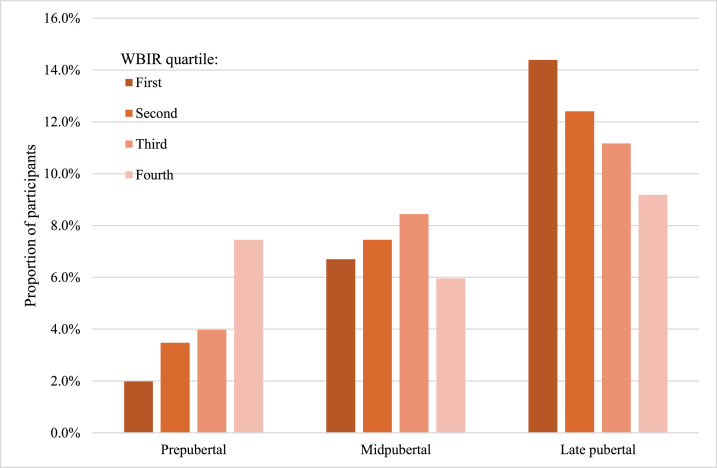
Pubertal stages and insulin resistance. Note: Higher age and advanced pubertal stage were associated with increased IR. Note: Participants in the first whole-body insulin resistance (WBIR) quartile have the highest IR.

Higher IR was present with advanced pubertal stages (Fig. 1). Q4 had the highest proportion of prepubertal participants (29.7 %; *p* < 0.001 for Q1 vs. Q4), whereas Q1 had the highest in late puberty (57.4 %; *p* = 0.045 for Q1 vs. Q4).

Compared with the full registry population, the 403 included children had marginally higher median weight (+2 kg) and BMI (+0.6 kg m⁻²), while age and height did not differ; fasting glucose and insulin were statistically—but not clinically—higher (**Supplementary Table 2**).

### 3.2 Glucose homeostasis

Significant differences were observed in glucose homeostasis and insulin sensitivity indices across WBIR quartiles (**Supplementary Table 3**), some expected due to the nature of the calculation of IR indices. Fasting glucose was highest in Q1 and slightly decreased with decreasing IR (*p* < 0.001). More pronounced differences were found in fasting insulin and post-load glucose and insulin levels (*p* < 0.001 for all). HbA1c differences were nonsignificant across WBIR groups (*p* = 0.257). We observed the expected absolute and relative elevations of insulin and glucose levels after glucose loading. In addition, these differences were most prominent in the most insulin-resistant group, particularly their absolute rise. Insulin at t120 (134.5 mE/L) demonstrated a specificity of 90.7 % and a sensitivity of 85.1 %, while glucose at t0 (6.0 mmol/L) had a specificity of 63.2 % and sensitivity of 82.2 % for identifying IR (those in Q1). The area under the curve (AUC) for insulin at t120 was 0.958, indicating excellent discriminative ability, while glucose at t120 showed moderate accuracy (AUC=0.778). Insulin levels at t120 and t0 (AUC=0.945) showed similar discriminative ability, while glucose at t120 improved over t0 (AUC=0.616).

Correlation analysis (**Supplementary Fig. 1**) revealed strong correlations among IR indices (Spearman's ρ≥0.50, *p* < 0.001), with SPISE (t0 and t120) correlating least with the others, likely due to differing calculation parameters. Similar trends were seen across WBIR groups (*p* < 0.001 for all indices), with Q4 having significantly lower IR compared to Q1 groups (*p* < 0.001 for all indices). Although the difference in SPISE between t0 and t120 was significant (*p* < 0.001, Wilcoxon rank-sum test), the correlations with WBIR were almost identical (ρ=0.504 vs. ρ=0.514), suggesting that t120 did not add any additional value in identifying IR.

### 3.3 Lipid homeostasis

Baseline HDL-C and TAG levels differed significantly between WBIR groups (*p* = 0.001 and *p* < 0.001, respectively), with TAG levels rising and HDL-C levels decreasing with higher IR (**Supplementary Table 4**). HDL-C differences were minor, while TAG levels at t120 were clinically meaningful (Q1: 1.30 (1.00–1.70) mmol/L vs. Q4: 0.70 (0.60–1.05) mmol/L; *p* < 0.001). Fasting TC and LDL-C levels were similar across groups (*p* > 0.050).

**Fig. 2:**
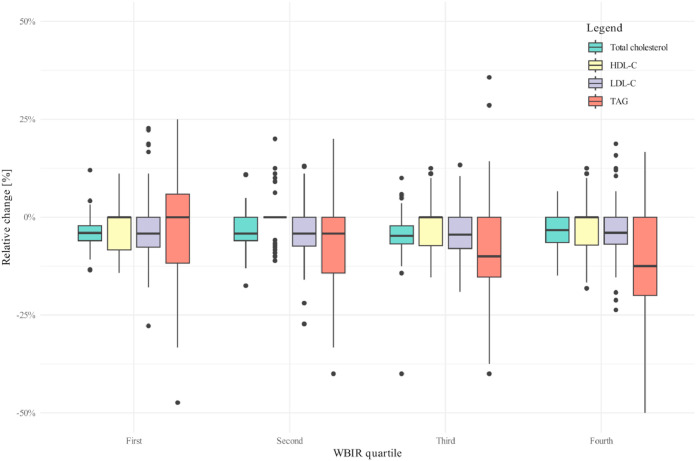
Association of whole body insulin resistance (WBIR) and relative change of total cholesterol, LDL cholesterol, HDL cholesterol and triglycerides (TAG) during glucose loading. Note: Participants in the first whole-body insulin resistance (WBIR) quartile have the highest IR.

Cholesterol levels remained largely unaffected by glucose loading, as the relative changes in TC, HDL-C, and LDL-C were minimal (−4,2 % for TC and LDL-C and 0 % for HDL-C; *p* > 0.050 for all). Differences after loading persisted for TAG with median levels of 1.30 (1.00–1.70) mmol/L in Q1 compared to 0.70 (0.50–1.00) mmol/L in Q4 (*p* < 0.001). TAG levels displayed a slight absolute reduction after glucose loading of 0.1 [0.2–0.0] in the whole cohort (Fig. 2); however, absolute reductions in TAG were comparable across WBIR groups (*p* = 0.400). At the same time, relative reductions in TAG levels were significant between groups and much more pronounced in the insulin-sensitive group (Q4), in which the largest reductions were observed (12.5 % [0–21.1]; *p* = 0.037 for in-group difference [Wilcoxon Rank-Sum Test]), while practically no changes were observed in TAG levels in the most insulin resistant group (*p* = 0.543 for in group difference [Wilcoxon Rank-Sum Test]). Spearman’s correlation analysis showed a moderate correlation between WBIR and fasting TAG (ρ=−0.474; *p* < 0.001), as well as TAG after glucose loading (ρ=−0.486; *p* < 0.001). Additional correlations between lipid profile parameters and WBIR are presented in **Supplementary Fig. 2**.

In the extended lipid panel, fasting ApoB and ApoB/ApoAI ratio increased with IR (*p* < 0.001), while ApoAI levels remained similar across groups (*p* = 0.374). Both ApoB and ApoAI levels were stable during eOGTT (relative reductions of −3.7 %, *p* = 0.983, and −2.2 %, *p* = 0.238, respectively).

### 3.4 Low-grade inflammation

hs-CRP showed a positive trend with IR across WBIR groups (*p* = 0.043; **Supplementary Table 5**), peaking in the most insulin-resistant group (1.38 [0.72–2.74]). Levels were similar in the other three groups (*p* = 0.664; **Supplementary Fig. 3**). Spearman's correlation with WBIR was weak but significant (ρ=−0.136; *p* = 0.012), suggesting increased low-grade inflammation in advanced IR states in the pediatric cohort."

### 3.5 Established insulin resistance biomarkers

Numerous established biomarkers were correlated with IR: TAG at t120 after OGTT, AST/ALT ratio, GGT, TAG/HDL-C ratio, and ApoB-to-LDL-C[21,[26], [27], [28], [29], [30]]. Our data in the pediatric cohort show a clear association and a level-dependence relationship with IR for all established biomarkers across WBIR groups (*p* < 0.001 for all; **Supplementary Table 6**).

Additionally, correlations (Spearman's) between these biomarkers were significant and revealed interconnected relationships (*p* = 0.035 for correlation between ApoB/LDL-C ratio and AST/ALT ratio; all other correlations: *p* < 0.001; **Supplementary Fig. 4**), except for the correlation between ApoB-to-LDL-C ratio and GGT (*p* = 0.130).

### 3.6 Metabolic parameters

**Fig. 3:**
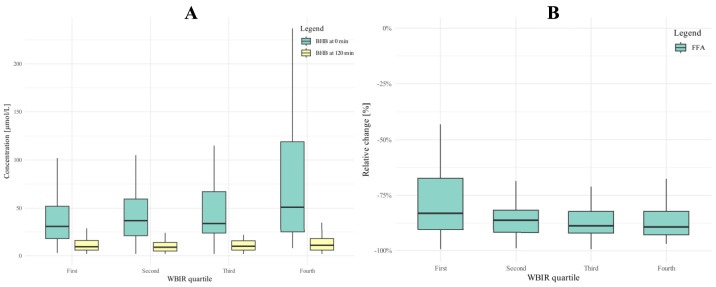
A) Beta-Hydroxybutyrate at 0 and 120 min across quartiles; B) Relative change in Free Fatty Acids (FFA) across the whole body insulin resistance (WBIR) quartiles.

Fasting BHB levels rose with decreasing IR, peaking in Q4 (*p* = 0.002), while no differences were observed after glucose loading (*p* = 0.500; Fig. 3**A**). Consequently, we observed that the absolute reductions in BHB (*p* = 0.003) after glucose intake increased with better insulin sensitivity (Table 2). The correlation (Spearman’s) between WBIR and fasting BHB, the absolute relative change in BHB were weak (ρ=0.233, ρ=−0.230, ρ=−0.165, respectively) but significant (*p* ≤ 0.001 for all three correlations).

**Table 2:** Beta-hydroxybutyrate, free fatty acids, and l-carnitine.

	Overall	Q1	Q2	Q3	Q4	p-value†^,^^a^	p-value§^,^^b^
**BHB at 0min, μmol/L**	37 (22–69)	31.5 (18–52.8)	37 (21–59.8)	34 (23.8–70.5)	51 (25–119.5)	0.002	<0.001
**BHB at 120min, μmol/L**	10 (6–16)	9.5 (6–16)	9 (5–14.8)	10 (6–16)	11 (6–18.5)	0.500	0.396
**Absolute Δ BHB, mmol/L**	−25 (−55 to −10)	−18.5 (−41 to −8)	−24.5 (−47.8 to −10)	−23.5 (−54 to −12)	−42 (−102 to −14)	0.003	<0.001
**Relative Δ BHB, %**	−74.3 (−88.4 to −51.3)	−70.0 (−86.7 to −42.7)	−76.0 (−88.8 to −52.6)	−72.1 (−88.9 to −50.0)	−79.8 (−91.1 to −56.5)	0.080	0.010
**FFA at 0min, mmol/L**	0.61 (0.34–0.91)	0.72 (0.31–0.92)	0.61 (0.37–0.88)	0.57 (0.34–0.90)	0.62 (0.24–0.98)	0.955	0.959
**FFA at 120min, mmol/L**	0.07 (0.06–0.12)	0.09 (0.07–0.22)	0.07 (0.06–0.11)	0.07 (0.04–0.09)	0.07 (0.05–0.09)	<0.001	0.001
**Absolute Δ FFA, mmol/L**	−0.53 (−0.80 to −0.27)	−0.59 (−0.75 to −0.25)	−0.53 (−0.75 to −0.28)	−0.51 (−0.81 to −0.28)	−0.52 (−0.91 to −0.20)	0.949	0.587^c^
**Relative Δ FFA, %**	−86.7 (−91.8 to −76.4)	−81.9 (−90.4 to −63.8)	−86.0 (−91.8 to −80.8)	−88.1 (−91.9 to −80.2)	−88.7 (−92.7 to −78.7)	0.068	0.021
**L-Carnitine, μmol/L**	45.5 (35.3–60)	48.5 (38.3–61.5)	49 (33–62)	47 (35–60)	45 (30.5–55.3)	0.353	0.080

Data are absolute frequency (proportion in %) and median (first quartile–third quartile).

**Legend:** Q – Quartile; BHB – Beta-Hydroxybutyrate; FFA – Free Fatty Acids; Δ – change between 0 min and 120 min (negative value means a reduction of the parameter during test).

**Footnotes:**.

† p-value for comparisons between all four groups;.

§ p-value for comparisons between Q1 and Q4;.

a - Kruskal-Wallis Test;.

b - Mann-Whitney Test;.

c - Independent Samples T-test. Post-hoc comparisons for significant results were performed using Dunn's test for Kruskal-Wallis, with p-values adjusted using the Benjamini-Hochberg method (significance set at adjusted *p* < 0.05). Detailed results for significant comparisons are provided below (parameter name: adjusted p-value for Q2 vs. Q1, Q3 vs. Q1, Q4 vs. Q1, Q3 vs. Q2, Q4 vs. Q2, Q4 vs. Q3): BHB at 0 min: 0.290, 0.129, 0.000, 0.587, 0.016, 0.051; Absolute Δ BHB: 0.333, 0.147, 0.001, 0.567, 0.020, 0.066; FFA at 120 min: 0.084, 0.001, 0.002, 0.084, 0.166, 0.679.

Levels of FFA also decreased during eOGTT. While fasting FFA levels were comparable in the four groups (*p* = 0.955), FFA at t120 displayed significant differences (*p* < 0.001). However, these differences were small in absolute terms, and the absolute and relative change in FFA was insignificant (*p* = 0.949 and 0.068, respectively; Fig. 3**B**). FFA after glucose loading and relative change in FFA were weakly correlated (Spearman’s) with WBIR (ρ=−0.195, *p* < 0.001; ρ=−0.159, *p* = 0.010). It is worth noting that the differences between groups could be diminished due to the methodology for determining FFA concentrations, as the method reports the result as semi-quantitative below the lower threshold of detectability.

Fasting l-carnitine levels did not differ significantly across quartiles (*p* = 0.353).

## 4 Discussion

Understanding IR in children and adolescents has become increasingly critical due to the global rise in pediatric obesity[1]. While IR has been extensively studied in adults, there is limited knowledge about the large-scale methods for early detection of children and adolescents with pronounced IR, as these have the greatest risk for developing T2D and cardiometabolic complications later in life.

Therefore, we conducted a cross-sectional clinical study aimed to identify metabolic biomarkers that allow for early detection of IR and obesity-associated metabolic complications in children and adolescents with overweight and obesity. While fasting steady-state markers are commonly used to define IR in children, our goal was to determine whether performing eOGTT, which captures the dynamic glucose response, provides additional information beyond parameters of glucose homeostasis and aids in identification of children with pronounced IR and metabolic abnormalities.

As obesity becomes an increasing public health issue, the implementation of eOGTT, which expands the conventional OGTT, could offer a clinically useful and cost-effective method for early identification of children and adolescents at higher risk for obesity-related complications without posing a significant burden on human resources, financial demands and logistics of the healthcare system[1,31]. This would allow for more precise monitoring and earlier intervention, helping to prevent future complications.

It is important to note that our comparison focuses on children and adolescents with overweight and obesity, with 98.5 % of participants exceeding the body weight threshold for obesity diagnosis, as IR is most pronounced in this population[16,32]. This adds further weight to our significant findings, as the observed differences would likely have been even more pronounced had we compared insulin-resistant subjects with healthy individuals.

Insulin-resistant participants in our cohort were significantly older and at more advanced pubertal stages. While aging increases the risk of T2D, likely due to its association with senile muscle dysfunction, this is not expected in the pediatric population[4]. Thus, the higher IR observed in older children and adolescents is likely attributed to pubertal progression, as expected with age. Pubertal IR is mediated by growth hormone, the production of which doubles during puberty, leading to increased total body lipolysis and fat oxidation[6,33].

We validated our cohort and the division into four WBIR groups using several approaches. First, we compared our results with other commonly used IR indices (**Supplementary Fig. 1**). Additionally, we assessed anthropometric indicators such as BMI Z-scores and waist-to-height ratios. We also evaluated basic glucose homeostasis parameters and cross-referenced our findings with established biomarkers of IR reported in the literature (**Supplementary Fig. 4**)[21,[26], [27], [28], [29], [30]]. These methods ensured a comprehensive validation of our cohort and provided robust support for the accuracy of our further analysis.

Regarding the study's aim, our findings indicate that measuring insulin and glucose levels after eOGTT is valuable for characterizing IR. These measurements exhibited better discriminative ability in identifying IR subjects compared to baseline measurements. Conversely, HbA1c proved to be an ineffective marker for IR, making its measurement redundant for diagnosing the condition. The lack of significant differences in HbA1c suggests that IR was adequately compensated by increased insulin secretion from pancreatic beta cells[34]. Notably, differences in glucose homeostasis across WBIR quartiles align with expectations due to the nature of the WBIR index calculations.

Lipid profile analysis showed no significant changes in cholesterol levels after eOGTT, likely due to the short 120-minute window. At baseline, HDL-C varied significantly across WBIR quartiles, while TC and LDL-C differences were insignificant. Low HDL-C in IR is commonly attributed to mechanisms such as increased TAG exchange from chylomicrons and very low-density lipoprotein with HDL-C, reduced lipoprotein lipase activity, enhanced HDL-C clearance due to increased hepatic triglyceride lipase, and decreased ApoAI synthesis and secretion[[35], [36], [37]].

In our study, TAG levels at t0 and t120 declined with higher insulin sensitivity, consistent with previous research[29,38]. Vossen et al. previously highlighted the importance of measuring TAG after eOGTT, showing that post-eOGTT change in TAG often correlated more strongly with IR markers than fasting TAG, suggesting it could be a more robust risk marker[39]. The observed TAG decline during eOGTT is likely mediated by insulin’s effects, as insulin lowers plasma TAG by reducing VLDL secretion from the liver and enhancing TAG clearance through lipoprotein lipase and remnant receptors[[40], [41], [42]]. A key clinical finding from our study is that glucose loading had practically no effect on TAG levels in the most insulin-resistant individuals, whereas greater TAG declines were observed with increasing insulin sensitivity. The absence of a TAG decline after glucose loading may serve as an indicator of insulin resistance.

The higher baseline ApoB levels in more insulin-resistant participants were expected, as insulin usually reduces ApoB secretion and enhances its clearance[43]. Also, association of baseline ApoB/ApoAI ratio with IR was consistent with previous findings[44]. ApoB and ApoAI levels remained relatively stable during the eOGTT, suggesting that measuring apolipoprotein levels after glucose loading does not offer additional insight into IR. However, our results remain important: since each ApoB-containing lipoprotein particle has a single ApoB molecule, ApoB concentration reflects the number of these particles in the blood[45]. The stable ApoB levels during the eOGTT imply that changes in TAG levels after glucose loading likely reflect variations in TAG mass rather than changes in the mass of lipoproteins transporting TAG.

The median hs-CRP in our cohort (1.38 [0.72–2.74] mg/L) was significantly higher than the 0.2 to 0.3 mg/L reported for the normal pediatric population, likely reflecting increased inflammation, possibly due to low-grade inflammation associated with obesity[46]. Additionally, peak hs-CRP was highest in the most insulin-resistant group, indicating more pronounced low-grade inflammation with advancing IR. The elevated hs-CRP levels may be partially explained by higher BMI, as previous studies have shown a correlation between higher BMI and increased CRP concentrations[47]. However, McLaughlin et al. demonstrated that CRP levels are linked to the degree of IR and hyperinsulinemia, independent of BMI[48].

Elevated FFA are crucial in obesity-related IR. In individuals with obesity, increased FFA release from larger fat tissues disrupts insulin’s ability to suppress lipolysis, leading to even higher blood FFA levels[49]. While acute FFA elevation impairs insulin-stimulated glucose uptake, reducing FFA levels can improve IR and related conditions[50,51]. Baseline FFA levels in our study showed no difference across varying degrees of IR, questioning the role of FFAs in IR and aligning with Karpe et al.'s view that elevated FFAs are not always a mediator of IR, as FFA release per kilogram of adipose tissue decreases with adipose expansion, often normalizing plasma FFA levels in many individuals with obesity[52]. Methodological challenges in measuring FFAs in our study should also be considered.

Our results indicate that performing an eOGTT and monitoring reductions in BHB provides additional insight into IR, as smaller reductions were observed with increasing IR Ketogenesis is associated with IR, with individuals with T2D requiring higher insulin levels to suppress ketone production[53]. Although elevated ketone bodies are linked to T2D, obesity complicates this relationship by lowering plasma BHB levels and reducing BHB oxidation[54,55]. This was evident in our study, where more insulin-resistant subjects had significantly lower baseline BHB levels, showing a strong negative correlation with WBIR, consistent with Garcia et al.'s findings[56].

### 4.1 Limitations

This study has several limitations. 1) Missing values were rare (< 4 %) and pattern-free, so complete-case analysis may have modestly reduced statistical power for secondary biomarkers. 2) Because 98 % of participants had obesity, the cohort carried an intrinsically higher burden of insulin resistance, dyslipidemia and low-grade inflammation; the absence of a lean comparison group limits generalizability and may exaggerate some effect sizes. 3) No 30-minute sample was drawn during the eOGTT, precluding assessment of first-phase insulin secretion; future work should include denser time-points. 4) Only small, clinically insignificant differences in BMI and fasting glycaemia separated included from non-included children, so major selection bias is unlikely, yet replication in an unselected pediatric sample is warranted. 5) The cross-sectional design precludes causal inference and follow-up of insulin-resistance trajectories. 6) Our single-center, ethnically homogeneous sample may limit external validity. 7) Reliance on surrogate indices (e.g., HOMA-IR, ISI-M) rather than glucose-clamp measurements introduces potential measurement error, and their accuracy across pubertal stages requires further validation[17,19].

## 5 Conclusion

In this study, we validated several established markers of IR, previously demonstrated in adults, within a pediatric cohort. We also showed that performing an eOGTT allows for better and earlier characterization of children and adolescents with obesity, at risk for obesity-related complications. Special attention should be given to post-eOGTT TAG levels, alongside insulin and glucose measurements. Additionally, elevated hs-CRP and lower baseline BHB were associated with more severe IR.

Central illustration

**Central illustration:**
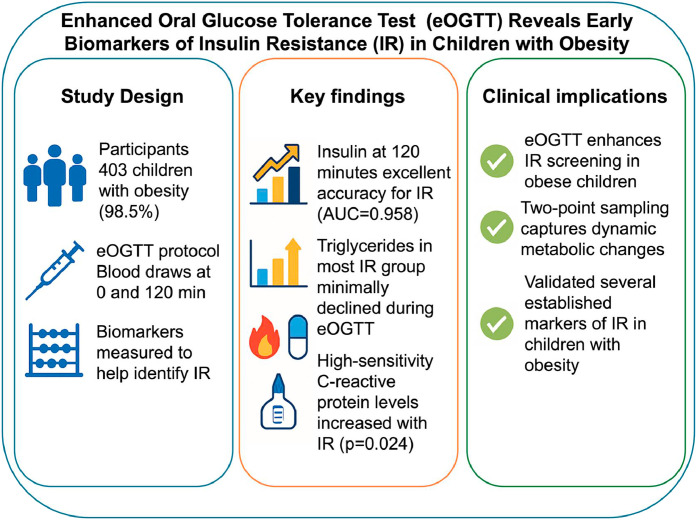
Enhanced oral glucose tolerance test (eOGTT) reveals early biomarkers of insulin resistance (IR) in children with obesity. The study included 403 children with obesity undergoing eOGTT with blood draws at 0 and 120 min. Key findings included excellent diagnostic performance of insulin at 120 min for IR identification (AUC=0.958), minimal triglyceride decline during eOGTT in insulin-resistant children, and higher hs-CRP levels with increasing IR. Clinical implications support eOGTT as an improved screening tool for IR in obese children, capturing dynamic metabolic changes and validating multiple biomarkers".

## Funding

This work was supported by the 10.13039/501100004329Slovenian Research and Innovation Agency (grant P3–0343). The funding organization had no role in the design and conduct of the study; collection, management, analysis, and interpretation of the data; preparation, review, or approval of the manuscript; and decision to submit the manuscript for publication.

## Ethics considerations

The study was approved by the Slovenian National Medical Ethics Committee (No. 29/02/13) and conducted according to the Declaration of Helsinki. Written informed consent was obtained from the child’s parents/caregivers prior to the inclusion of children and adolescents into the study.

## Data availability

Data is available upon request from the corresponding author.

## Originality of content

We confirm all information and materials in the manuscript are original.

## Author agreement statement

1. **Originality**: This **manuscript** represents original work, has not been previously published, and is not currently under consideration for publication elsewhere.
2. **Responsibility**: The **authors** take full responsibility for the integrity and accuracy of the data presented.

## CRediT authorship contribution statement

**Urh Groselj:** Writing – review & editing, Supervision, Methodology, Conceptualization. **Jan Kafol:** Writing – review & editing, Writing – original draft, Visualization, Methodology, Investigation, Formal analysis, Data curation, Conceptualization. **Jaka Sikonja:** Writing – review & editing, Writing – original draft, Methodology, Investigation, Formal analysis, Data curation, Conceptualization. **Matej Mlinaric:** Writing – review & editing, Investigation, Data curation. **Robert Sket:** Writing – review & editing, Visualization, Validation, Formal analysis. **Ziga Iztok Remec:** Writing – review & editing, Validation, Formal analysis. **Jernej Kovac:** Writing – review & editing, Validation, Methodology. **Ana Drole Torkar:** Writing – review & editing, Investigation, Data curation. **Jasna Suput Omladic:** Writing – review & editing, Investigation. **Barbka Repic Lampret:** Writing – review & editing, Validation, Data curation. **Tadej Battelino:** Writing – review & editing, Supervision, Funding acquisition. **Primoz Kotnik:** Writing – review & editing, Supervision, Methodology, Conceptualization.

## Declaration of competing interest

The authors declare that they have no known competing financial interests or personal relationships that could have appeared to influence the work reported in this paper.

## Appendix Supplementary materials

Image, application 1

## References

[1] Lobstein T., Jackson-Leach R., Moodie M.L. (2015). Child and adolescent obesity: part of a bigger picture. Lancet.

[2] Di Cesare M., Soric M., Bovet P. (2019). The epidemiological burden of obesity in childhood: a worldwide epidemic requiring urgent action. BMC Med.

[3] Li M., Chi X., Wang Y., Setrerrahmane S., Xie W., Xu H. (2022). Trends in insulin resistance: insights into mechanisms and therapeutic strategy. Signal Transduct Target Ther.

[4] Shou J., Chen P.J., Xiao W.H. (2020). Mechanism of increased risk of insulin resistance in aging skeletal muscle. Diabetol Metab Syndr.

[5] Dahlgren J. (2006). Pregnancy and insulin resistance. Metab Syndr Relat Disord.

[6] Hannon T.S., Janosky J., Arslanian S.A. (Dec 2006). Longitudinal study of physiologic insulin resistance and metabolic changes of puberty. Pediatr Res.

[7] Ahmed B., Sultana R., Greene M.W. (May 2021). Adipose tissue and insulin resistance in obese. Biomed Pharmacother.

[8] Weir G.C., Bonner-Weir S. (Dec 2004). Five stages of evolving beta-cell dysfunction during progression to diabetes. Diabetes.

[9] Ghosh C., Mukhopadhyay P., Ghosh S., Pradhan M. (2015). Insulin sensitivity index (ISI0, 120) potentially linked to carbon isotopes of breath CO2 for pre-diabetes and type 2 diabetes. Sci Rep.

[10] Muniyappa R., Lee S., Chen H., Quon M.J. (Jan 2008). Current approaches for assessing insulin sensitivity and resistance in vivo: advantages, limitations, and appropriate usage. Am J Physiol Endocrinol Metab.

[11] DeFronzo R.A., Tobin J.D., Andres R. (Sep 1979). Glucose clamp technique: a method for quantifying insulin secretion and resistance. Am J Physiol.

[12] Gastaldelli A. (Aug 2022). Measuring and estimating insulin resistance in clinical and research settings. Obesity (Silver Spring).

[13] Ortiz-Martinez M., Gonzalez-Gonzalez M., Martagon A.J., Hlavinka V., Willson R.C., Rito-Palomares M. (Mar 2022). Recent developments in biomarkers for diagnosis and screening of type 2 diabetes mellitus. Curr Diab Rep.

[14] Cole T.J., Freeman J.V., Preece M.A. (1998). British 1990 growth reference centiles for weight, height, body mass index and head circumference fitted by maximum penalized likelihood. Stat Med.

[15] Pan H., Cole T.J. LMSgrowth, a Microsoft Excel add-In to access growth references based on the LMS method, version 2.77. Accessed 10.6.2024.

[16] Kumar S., Kelly A.S. (Feb 2017). Review of childhood obesity: from epidemiology, etiology, and comorbidities to clinical assessment and treatment. Mayo Clin Proc.

[17] Matthews D.R., Hosker J.P., Rudenski A.S., Naylor B.A., Treacher D.F., Turner R.C. (Jul 1985). Homeostasis model assessment: insulin resistance and beta-cell function from fasting plasma glucose and insulin concentrations in man. Diabetologia.

[18] DeFronzo R.A., Matsuda M. (Jul 2010). Reduced time points to calculate the composite index. Diabetes Care.

[19] Matsuda M., DeFronzo R.A. (Sep 1999). Insulin sensitivity indices obtained from oral glucose tolerance testing: comparison with the euglycemic insulin clamp. Diabetes Care.

[20] Barchetta I., Dule S., Bertoccini L. (Jan 2022). The single-point insulin sensitivity estimator (SPISE) index is a strong predictor of abnormal glucose metabolism in overweight/obese children: a long-term follow-up study. J Endocrinol Invest.

[21] Paulmichl K., Hatunic M., Hojlund K. (Sep 2016). Modification and validation of the triglyceride-to-HDL cholesterol ratio as a surrogate of insulin sensitivity in white juveniles and adults without diabetes mellitus: the single point insulin sensitivity estimator (SPISE). Clin Chem.

[22] Katz A., Nambi S.S., Mather K. (Jul 2000). Quantitative insulin sensitivity check index: a simple, accurate method for assessing insulin sensitivity in humans. J Clin Endocrinol Metab.

[23] Lorenzo C., Haffner S.M., Stancakova A., Kuusisto J., Laakso M. (Feb 2015). Fasting and OGTT-derived measures of insulin resistance as compared with the euglycemic-hyperinsulinemic clamp in nondiabetic Finnish offspring of type 2 diabetic individuals. J Clin Endocrinol Metab.

[24] Selimoglu H., Duran C., Kiyici S. (Oct 2009). Comparison of composite whole body insulin sensitivity index derived from mixed meal test and oral glucose tolerance test in insulin resistant obese subjects. Endocrine.

[25] VA Malagon-Soriano, Ledezma-Forero A.J., Espinel-Pachon C.F. (2024). Surrogate indices of insulin resistance using the Matsuda index as reference in adult men-a computational approach. Front Endocrinol (Lausanne).

[26] Larcher B., Maechler M., Sprenger L. (2023). 260-OR: the ApoB/LDL-C ratio predicts major cardiovascular events in cardiovascular disease patients independent of type 2 diabetes status. Diabetes.

[27] Pantoja-Torres B., Toro-Huamanchumo C.J., Urrunaga-Pastor D. (2019). High triglycerides to HDL-cholesterol ratio is associated with insulin resistance in normal-weight healthy adults. Diabetes Metab Syndr.

[28] Thamer C., Tschritter O., Haap M. (Apr 2005). Elevated serum GGT concentrations predict reduced insulin sensitivity and increased intrahepatic lipids. Horm Metab Res.

[29] Ma M., Liu H., Yu J. (2020). Triglyceride is independently correlated with insulin resistance and islet beta cell function: a study in population with different glucose and lipid metabolism states. Lipids Health Dis.

[30] Minato-Inokawa S., Tsuboi-Kaji A., Honda M. (2023). Associations of alanine aminotransferase/aspartate aminotransferase with insulin resistance and beta-cell function in women. Sci Rep.

[31] Icks A., Haastert B., Gandjour A. (Sep 2004). Cost-effectiveness analysis of different screening procedures for type 2 diabetes: the KORA Survey 2000. Diabetes Care.

[32] Chiarelli F., Marcovecchio M.L. (Dec 2008). Insulin resistance and obesity in childhood. Eur J Endocrinol.

[33] Saenger P. (2003). Dose effects of growth hormone during puberty. Horm Res.

[34] Kasuga M. (Jul 2006). Insulin resistance and pancreatic beta cell failure. J Clin Invest.

[35] de Vries R., Borggreve S.E., Dullaart R.P. (2003). Role of lipases, lecithin:cholesterol acyltransferase and cholesteryl ester transfer protein in abnormal high density lipoprotein metabolism in insulin resistance and type 2 diabetes mellitus. Clin Lab.

[36] Garg A. (Apr 1996). Insulin resistance in the pathogenesis of dyslipidemia. Diabetes Care.

[37] Bjornstad P., Eckel R.H. (2018). Pathogenesis of lipid disorders in insulin resistance: a brief review. Curr Diab Rep.

[38] Cho J., Hong H., Park S., Kim S., Kang H. (2017). Insulin resistance and its association with metabolic syndrome in Korean children. Biomed Res Int.

[39] Vossen M., Todter K., Altenburg C., Beisiegel U., Scheja L. (Jul 2011). Plasma triglycerides after oral glucose load specifically associate with metabolic risk markers in healthy type 2 diabetes offspring. Atherosclerosis.

[40] Pelikanova T., Kohout M., Base J. (1991). Effect of acute hyperinsulinemia on fatty acid composition of serum lipids in non-insulin-dependent diabetics and healthy men. Clin Chim Acta.

[41] Adiels M., Olofsson S.O., Taskinen M.R., Boren J. (Jul 2008). Overproduction of very low-density lipoproteins is the hallmark of the dyslipidemia in the metabolic syndrome. Arterioscler Thromb Vasc Biol.

[42] Laatsch A., Merkel M., Talmud P.J., Grewal T., Beisiegel U., Heeren J. (May 2009). Insulin stimulates hepatic low density lipoprotein receptor-related protein 1 (LRP1) to increase postprandial lipoprotein clearance. Atherosclerosis.

[43] Haas M.E., Attie A.D., Biddinger S.B. (Aug 2013). The regulation of ApoB metabolism by insulin. Trends Endocrinol Metab.

[44] Sierra-Johnson J., Romero-Corral A., Somers V.K. (Nov 2007). ApoB/apoA-I ratio: an independent predictor of insulin resistance in US non-diabetic subjects. Eur Heart J.

[45] Sniderman A.D., Thanassoulis G., Glavinovic T. (2019). Apolipoprotein B particles and cardiovascular disease: a narrative review. JAMA Cardiol.

[46] Schlenz H., Intemann T., Wolters M. (Sep 2014). C-reactive protein reference percentiles among pre-adolescent children in Europe based on the IDEFICS study population. Int J Obes (Lond).

[47] Visser M., Bouter L.M., McQuillan G.M., Wener M.H., Harris T.B. (1999). Elevated C-reactive protein levels in overweight and obese adults. JAMA.

[48] McLaughlin T., Abbasi F., Lamendola C. (2002). Differentiation between obesity and insulin resistance in the association with C-reactive protein. Circulation.

[49] Boden G. (Apr 2011). Obesity, insulin resistance and free fatty acids. Curr Opin Endocrinol Diabetes Obes.

[50] Boden G., Jadali F., White J. (Sep 1991). Effects of fat on insulin-stimulated carbohydrate metabolism in normal men. J Clin Invest.

[51] Santomauro A.T., Boden G., Silva M.E. (Sep 1999). Overnight lowering of free fatty acids with Acipimox improves insulin resistance and glucose tolerance in obese diabetic and nondiabetic subjects. Diabetes.

[52] Karpe F., Dickmann J.R., Frayn K.N. (Oct 2011). Fatty acids, obesity, and insulin resistance: time for a reevaluation. Diabetes.

[53] Singh B.M., Krentz A.J., Nattrass M. (Apr 1993). Insulin resistance in the regulation of lipolysis and ketone body metabolism in non-insulin dependent diabetes is apparent at very low insulin concentrations. Diabetes Res Clin Pract.

[54] Szili-Torok T., de Borst M.H., Garcia E. (2023). Fasting ketone bodies and incident type 2 diabetes in the general population. Diabetes.

[55] Soeters M.R., Sauerwein H.P., Faas L. (Jul 2009). Effects of insulin on ketogenesis following fasting in lean and obese men. Obesity (Silver Spring).

[56] Garcia E., Shalaurova I., Matyus S.P. (Jan 23 2020). Ketone bodies are mildly elevated in subjects with type 2 diabetes mellitus and are inversely associated with insulin resistance as measured by the lipoprotein insulin resistance index. J Clin Med.

