# Supplementary material for: Enhanced oral glucose tolerance test for early detection of insulin resistance and metabolic complications in children with obesity
Source: Am J Prev Cardiol. 2025 Jun 4;23:101016. doi: 10.1016/j.ajpc.2025.101016 (PMC12205605; doi:10.1016/j.ajpc.2025.101016)
Supplement: Supplementary file 1 [file mmc1.docx]

Supplementary Materials

Table of Contents

[METHODS 2](#_Toc183718563)

[Inclusion criteria 2](#_Toc183718564)

[Exclusion criteria 2](#_Toc183718565)

[Pubertal stage assessment 3](#_Toc183718566)

[Insulin resistance indices calculation 3](#_Toc183718567)

[RESULTS 4](#_Toc183718568)

[Glucose homeostasis 4](#_Toc183718569)

[Correlation between IR indices 6](#_Toc183718570)

[Lipid homeostasis 7](#_Toc183718571)

[Correlation between lipid homeostasis parameters and WBIR 9](#_Toc183718572)

[Low-grade inflammation 10](#_Toc183718573)

[Established insulin resistance markers 12](#_Toc183718574)

[REFERENCES 14](#_Toc183718575)

# METHODS

## Inclusion criteria

Supplementary Table 1: Complete set of laboratory measurements required for inclusion in the study.

| **Baseline (t_0 min_)** | **After glucose loading (t_120 min_)** |
| --- | --- |
| - glucose, - insulin, - total cholesterol (TC), - high-density lipoprotein cholesterol (HDL-C), - low-density lipoprotein cholesterol (LDL-C), - triglycerides (TAG), - apolipoprotein AI (ApoAI), - apolipoprotein B (ApoB) | - glucose, - insulin, - total cholesterol (TC), - high-density lipoprotein cholesterol (HDL-C), - low-density lipoprotein cholesterol (LDL-C), - triglycerides (TAG), - apolipoprotein AI (ApoAI), - apolipoprotein B (ApoB) |
| - alanine transaminase (ALT), - aspartate aminotransferase (AST), - gamma-glutamyl transpeptidase (GGT), - high-sensitivity C-reactive protein (hs-CRP) |  |

## Exclusion criteria

The exclusion criteria were:

- Treatment with metformin, insulin, glucocorticoids, or growth hormone analogs;
- Or the presence of any of the following comorbidities:
  - polycystic ovary syndrome,
  - pituitary gland disorder or hypopituitarism,
  - hypothyroidism (thyroid-stimulating hormone [TSH] > 5.0 mU/L regardless of levothyroxine treatment),
  - genetic syndrome (such as Turner, Down, Williams, von Hippel-Lindau, DiGeorge, etc.),
  - sex development disorder (hypogonadism, precocious puberty, delayed puberty, testicular hypofunction, primary amenorrhea),
  - the disorder of childhood growth (microcephaly, short stature, delayed development, small for gestational age),
  - inborn error of metabolism,
  - neurological dysfunction (epilepsy, spastic tetraplegia, extrapyramidal disorder, cerebrovascular insult), and
  - other comorbidities that could affect IR (toxic epidermal necrolysis, Chiari malformation, pineal gland cyst, nephrectomy).

Supplementary Table 2: Baseline anthropometric and biochemical characteristics of included children (n = 403) versus the full registry population (n = 1 664): median (Q1–Q3) and Mann–Whitney U p-values.

| Variable | Included (n=403) | All patients (n=1 664) | p-value |
| --- | --- | --- | --- |
| Age (years) | 13.18 (10.45–15.21) | 13.25 (10.24–15.73) | 0.757 |
| Height (cm) | 162.0 (151.4–171.2) | 161.7 (149.0–170.0) | 0.075 |
| Weight (kg) | 78.5 (62.1–94.9) | 76.1 (58.7–92.2) | 0.038 |
| BMI (kg/m²) | 29.43 (26.47–33.17) | 28.87 (25.67–32.78) | 0.045 |
| Glucose 0 min (mmol/L) | 4.60 (4.40–4.90) | 4.70 (4.50–5.00) | <0.001 |
| Insulin 0 min (mE/L) | 15.1 (8.8–22.2) | 12.8 (8.0–19.9) | 0.001 |

## Pubertal stage assessment

Pubertal stages were determined using Tanner classification: pubic (pubarche – P) and axillary (adrenarche – A) hair growth, breast development (in females; thelarche – T), and testicular volume (in males; TV). TV was measured by an orchidometer. “Prepubertal” stage was defined as Tanner stage P1A1T1 (in females), and P1A1 and TV < 4 ml (in males); “late or finished pubertal” stage as P ≥ 4 or A3 or T ≥ 4 (in females) and as P ≥ 4 or A3 or VT > 15 ml (in males); “midpubertal” stage as stages between “prepubertal” and “late or finished pubertal”.^1,2^

## Insulin resistance indices calculation

Homeostatic Model Assessment for Insulin Resistance (HOMA-IR) estimates insulin resistance from fasting glucose and fasting insulin.^3^

$$HOMA-IR =\frac{fasting glucose \left[ mmol/L \right] \times fasting insulin \left[ mU/L \right]}{22,5}$$

Matsuda Insulin Sensitivity Index (ISI-M) estimates insulin resistance from glucose and insulin during OGTT. A simplified ISI-M equation that requires measurements at only two-time points was used.^4,5^

$$ISI-M =\frac{10.000}{\sqrt{G_{0} [mg/dL] \times I_{0} [\mu U/mL] \times G_{120} [mg/dL] \times I_{120} [\mu U/mL]}}$$

Single Point Insulin Sensitivity Estimator (SPISE) estimates insulin resistance from HDL-C, TAG, and body mass index (BMI).^6,7^ We calculated SPISE at baseline and after glucose loading.

$$SPISE =600 \times\frac{{HDL-C}^{0.185}[\frac{mg}{dL}]}{{TAG}^{0.2} [mg/dL] \times{ITM}^{1.338} [kg/m^{2}]}$$

Quantitative insulin sensitivity check index (QUICKI) estimates insulin resistance from fasting glucose and insulin.^8^

$QUICKI = \frac{1}{log(I_{0}) [\mu U/mL] + log(G_{0}) [mg/dL]}$

# RESULTS

## Glucose homeostasis

Supplementary Table 3: Glucose homeostasis.

|  | **Overall** | **Q1*** | **Q2** | **Q3** | **Q4** | **p-value**†**^a^** | **p-value**§ |
| --- | --- | --- | --- | --- | --- | --- | --- |
| **Glucose at 0 min, mmol/l** | 4.60 (4.40–4.90) | 4.70 (4.45–5.05) | 4.60 (4.40–4.85) | 4.60 (4.40–4.88) | 4.50 (4.30–4.70) | <0.001 | <0.001^b^ |
| **Insulin at 0 min, mU/L** | 15.1 (8.8–22.2) | 29.1 (22.2–35.8) | 17.4 (14.6–20.2) | 12.1 (9.0–14.8) | 6.5 (4.8–8.8) | <0.001 | <0.001^b^ |
| **Glucose at 120 min, mmol/L** | 6.00 (5.20–6.70) | 6.70 (6.20–7.70) | 6.30 (5.60–6.95) | 5.60 (5.00–6.20) | 5.20 (4.45–5.85) | <0.001 | <0.001^c^ |
| **Insulin at 120 min, mU/L** | 89.9 (54.8–146.7) | 196 (154–271) | 113.7 (95.8–137.5) | 68.0 (58.4–86.6) | 41.8 (28.7–52.3) | <0.001 | <0.001^b^ |
| **Absolute Δ Glucose, mmol/L** | 1.30 (0.60–2.10) | 2.10 (1.40–2.90) | 1.70 (1.00–2.25) | 0.90 (0.40–1.68) | 0.80 (0.00–1.35) | <0.001 | <0.001^c^ |
| **Relative Δ Glucose, %** | 29.3 (13.0–46.7) | 41.7 (29.5–60.0) | 35.0 (22.1–48.4) | 20.0 (9.3–37.1) | 16.3 (0.0–29.7) | <0.001 | <0.001^c^ |
| **Absolute Δ Insulin, mU/L** | 75.1 (41.0–122.4) | 172.9 (118.8–231.8) | 96.1 (75.7–121.4) | 56.0 (42.8–77.8) | 35.4 (20.4–46.0) | <0.001 | <0.001^b^ |
| **Relative Δ Insulin, %** | 540 (355–842) | 584 (393–956) | 567 (403–808) | 467 (309–793) | 521 (312–873) | 0.210 | 0.188^b^ |
| **HbA1c, %** | 5.2 (5.0–5.4) | 5.3 (5.0–5.4) | 5.3 (5.0–5.4) | 5.2 (5.0–5.4) | 5.2 (5.0–5.3) | 0.257 | 0.125^b^ |
| **ISI-M** | 2.90 (1.87–5.06) | 1.38 (1.04–1.58) | 2.36 (2.05–2.62) | 3.89 (3.23–4.40) | 6.93 (5.85–9.24) | <0.001 | <0.001^b^ |
| **HOMA-IR** | 3.03 (1.80–4.57) | 5.96 (4.60–7.85) | 3.48 (2.94–4.23) | 2.47 (1.82–3.01) | 1.28 (0.93–1.77) | <0.001 | <0.001^b^ |
| **QUICKI** | 0.32 (0.31–0.35) | 0.30 (0.29–0.31) | 0.32 (0.31–0.33) | 0.33 (0.32–0.35) | 0.37 (0.35–0.39) | <0.001 | <0.001^b^ |
| **SPISE at 0 min** | 5.26 (4.32–6.34) | 4.25 (3.52–5.19) | 5.01 (4.28–5.89) | 5.51 (4.74–6.44) | 6.41 (5.30–7.33) | <0.001 | <0.001 |
| **SPISE at 120 min** | 5.32 (4.32 - 6.40) | 4.26 (3.53 - 5.26) | 5.07 (4.30 - 5.89) | 5.52 (4.77 - 6.41) | 6.61 (5.39 - 7.50) | <0.001 | <0.001 |
| Data are absolute frequency (proportion in %) and median (first quartile–third quartile).  **Legend:** Q – Quartile; ISI-M – Matsuda Insulin Sensitivity Index; HOMA-IR – Homeostasis Model Assessment for Insulin Resistance; QUICKI – Quantitative insulin sensitivity check index; SPISE – Single Point Insulin Sensitivity Estimator; Δ – change between 0 min and 120 min (negative value means a reduction of the parameter during test).  **Footnotes:** *Q1 represents individuals with the highest insulin resistance; † p-value for comparisons between all four groups; § p-value for comparisons between Q1 and Q4; ^a^ – Kruskal-Wallis Test; ^b^ – Mann-Whitney Test; ^c^ – Independent Samples T-test.  Post-hoc comparisons for significant results were performed using Dunn's test for Kruskal-Wallis, with p-values adjusted using the Benjamini-Hochberg method (significance set at adjusted p < 0.05). Detailed results for significant comparisons are provided below (parameter name: adjusted p-value for Q2 vs. Q1, Q3 vs. Q1, Q4 vs. Q1, Q3 vs. Q2, Q4 vs. Q2, Q4 vs. Q3): Glucose at 0 min: 0.068, 0.045, 0.000, 0.703, 0.040, 0.056; Insulin at 0 min: <0.001, <0.001, <0.001, <0.001, <0.001, <0.001; Glucose at 120 min: 0.002, <0.001, <0.001, <0.001, <0.001, 0.004; Insulin at 120 min: <0.001, <0.001, <0.001, <0.001, <0.001, <0.001; Absolute Δ Glucose: 0.027, <0.001, <0.001, <0.001, <0.001, 0.029; Relative Δ Glucose: 0.046, <0.001, <0.001, <0.001, <0.001, 0.046; Absolute Δ Insulin: <0.001, <0.001, <0.001, <0.001, <0.001, <0.001; ISI-M: <0.001, <0.001, <0.001, <0.001, <0.001, <0.001; HOMA-IR: <0.001, <0.001, <0.001, <0.001, <0.001, <0.001; QUICKI: <0.001, <0.001, <0.001, <0.001, <0.001, <0.001; SPISE at 0 min: <0.001, <0.001, <0.001, 0.012, <0.001, 0.001; SPISE at 120 min: <0.001, <0.001, <0.001, 0.017, <0.001, <0.001. | | | | | | | |

### Correlation between IR indices


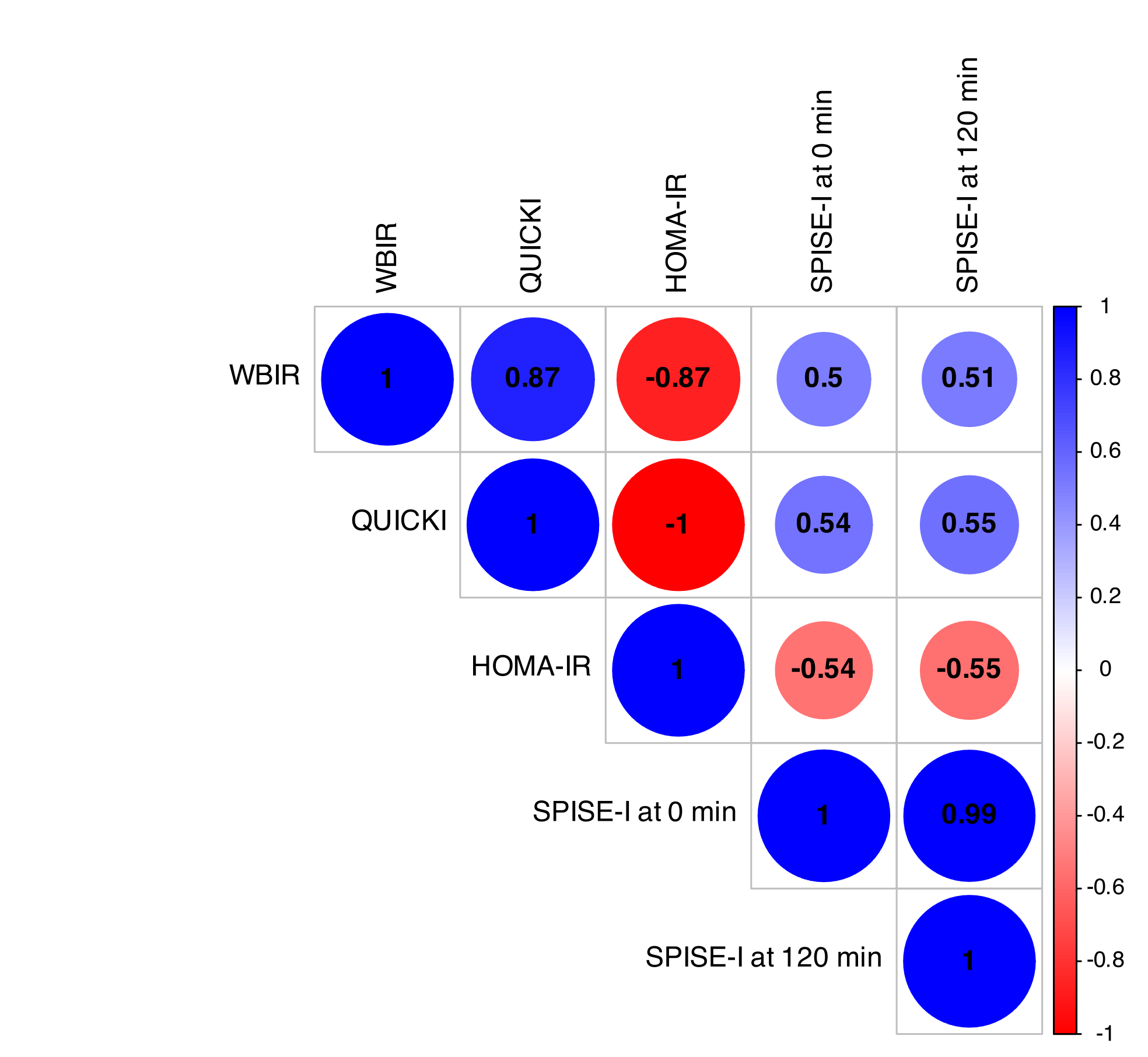


Supplementary Figure 1: Correlation analysis between four commonly used IR indices. Note: Numbers in the circles represent Spearman's rank correlation coefficients.

## Lipid homeostasis

Supplementary Table 4: Lipid profile.

|  | **Overall** | **Q1*** | **Q2** | **Q3** | **Q4** | **p-value**†**^a^** | **p-value**§**^b^** |
| --- | --- | --- | --- | --- | --- | --- | --- |
| **TC at 0 min, mmol/L** | 4.10 (3.60–4.70) | 4.20 (3.70–4.85) | 4.20 (3.65–4.75) | 4.05 (3.60–4.58) | 4.00 (3.55–4.50) | 0.072 | 0.014 |
| **HDL-C at 0 min, mmol/L** | 1.10 (1.00–1.30) | 1.00 (0.90–1.20) | 1.10 (1.00–1.30) | 1.20 (1.00–1.30) | 1.20 (1.00–1.40) | 0.001 | <0.001 |
| **LDL-C at 0 min, mmol/L** | 2.50 (2.10–3.00) | 2.60 (2.25–3.10) | 2.50 (2.20–3.00) | 2.40 (2.10–2.90) | 2.40 (2.00–2.85) | 0.121 | 0.028 |
| **TAG at 0 min, mmol/L** | 1.10 (0.80–1.40) | 1.30 (1.00–1.70) | 1.10 (0.90–1.50) | 1.00 (0.73–1.30) | 0.70 (0.60–1.05) | <0.001 | <0.001 |
| **TC at 120 min, mmol/L** | 4.00 (3.50–4.50) | 4.10 (3.50–4.70) | 4.00 (3.60–4.50) | 4.00 (3.40–4.40) | 3.80 (3.40–4.30) | 0.147 | 0.050 |
| **HDL-C at 120 min, mmol/L** | 1.10 (1.00–1.30) | 1.00 (0.90–1.20) | 1.10 (0.90–1.30) | 1.10 (1.00–1.20) | 1.20 (1.00–1.40) | <0.001 | <0.001 |
| **LDL-C at 120 min, mmol/L** | 2.40 (2.00–2.90) | 2.50 (2.10–3.00) | 2.40 (2.10–2.85) | 2.30 (1.93–2.80) | 2.30 (1.90–2.75) | 0.099 | 0.025 |
| **TAG at 120 min, mmol/L** | 1.00 (0.70–1.40) | 1.30 (1.00–1.70) | 1.10 (0.80–1.40) | 0.90 (0.70–1.28) | 0.70 (0.50–1.00) | <0.001 | <0.001 |
| **Absolute Δ TC, mmol/L** | -0.20 (-0.30 to -0.10) | -0.20 (-0.30 to -0.10) | -0.20 (-0.30 to 0.00) | -0.20 (-0.30 to -0.10) | -0.10 (-0.25 to 0.00) | 0.532 | 0.303 |
| **Relative Δ TC, %** | -4.2 (-6.4 to -1.9) | -4.1 (-6.2 to -2.2) | -4.2 (-6.0 to 0.0) | -4.8 (-6.8 to -2.1) | -3.3 (-6.5 to 0.0) | 0.598 | 0.496 |
| **Absolute Δ HDL-C, mmol/L** | 0.00 (-0.10 to 0.00) | 0.00 (-0.10 to 0.00) | 0.00 (-0.05 to 0.00) | 0.00 (-0.10 to 0.00) | 0.00 (-0.10 to 0.00) | 0.500 | 0.360 |
| **Relative Δ HDL-C, %** | 0.0 (-7.1 to 0.0) | 0.0 (-8.3 to 0.0) | 0.0 (-2.9 to 0.0) | 0.0 (-7.6 to 0.0) | 0.0 (-7.1 to 0.0) | 0.329 | 0.132 |
| **Absolute Δ LDL-C, mmol/L** | -0.10 (-0.20 to 0.00) | -0.10 (-0.20 to 0.00) | -0.10 (-0.20 to 0.00) | -0.10 (-0.20 to 0.00) | -0.10 (-0.20 to 0.00) | 0.890 | 0.469 |
| **Relative Δ LDL-C, %** | -4.2 (-7.7 to 0.0) | -4.2 (-7.8 to 0.0) | -4.2 (-7.5 to 0.0) | -4.4 (-8.0 to 0.0) | -4.0 (-7.4 to 0.0) | 0.955 | 0.710 |
| **Absolute Δ TAG, mmol/L** | -0.10 (-0.20 to 0.00) | 0.00 (-0.20 to 0.10) | -0.10 (-0.20 to 0.00) | -0.10 (-0.20 to 0.00) | -0.10 (-0.20 to 0.00) | 0.400 | 0.117 |
| **Relative Δ TAG, %** | -6.7 (-15.4 to 0.0) | 0.0 (-11.8 to 6.2) | -4.2 (-14.3 to 0.0) | -9.2 (-15.3 to 0.0) | -12.5 (-21.1 to 0.0) | 0.002 | <0.001 |
| **ApoAI at 0 min, g/L** | 1.31 (1.20–1.42) | 1.29 (1.20–1.41) | 1.30 (1.18–1.42) | 1.33 (1.24–1.46) | 1.29 (1.20–1.42) | 0.374 | 0.457^c^ |
| **ApoB at 0 min, g/L** | 0.76 (0.63–0.88) | 0.81 (0.69–0.95) | 0.78 (0.65–0.88) | 0.72 (0.59–0.86) | 0.71 (0.58–0.85) | <0.001 | <0.001 |
| **ApoB/ApoAI at 0 min** | 0.58 (0.48–0.68) | 0.64 (0.53–0.78) | 0.58 (0.51–0.68) | 0.56 (0.46–0.65) | 0.55 (0.46–0.66) | <0.001 | <0.001 |
| **ApoAI at 120 min, g/L** | 1.28 (1.18–1.39) | 1.27 (1.16–1.37) | 1.29 (1.17–1.39) | 1.30 (1.22–1.43) | 1.29 (1.18–1.43) | 0.345 | 0.321 |
| **ApoB at 120 min, g/L** | 0.73 (0.60–0.85) | 0.79 (0.66–0.92) | 0.75 (0.64–0.84) | 0.71 (0.57–0.82) | 0.68 (0.56–0.79) | <0.001 | <0.001 |
| **ApoB/ApoAI at 120 min** | 0.57 (0.47–0.68) | 0.62 (0.54–0.77) | 0.57 (0.50–0.66) | 0.57 (0.44–0.65) | 0.53 (0.45–0.64) | <0.001 | <0.001 |
| **Absolute Δ ApoAI, g/L** | -0.03 (-0.06 to 0.00) | -0.03 (-0.06 to 0.00) | -0.03 (-0.06 to 0.01) | -0.03 (-0.06 to 0.00) | -0.02 (-0.05 to 0.01) | 0.197 | 0.086 |
| **Relative Δ ApoAI, %** | -2.2 (-4.2 to 0.0) | -2.4 (-4.7 to 0.0) | -2.4 (-4.0 to 0.6) | -2.5 (-4.3 to 0.0) | -1.2 (-3.9 to 0.8) | 0.238 | 0.097 |
| **Absolute Δ ApoB, g/L** | -0.03 (-0.05 to -0.01) | -0.03 (-0.05 to -0.01) | -0.03 (-0.05 to -0.01) | -0.03 (-0.04 to 0.00) | -0.02 (-0.05 to -0.01) | 0.890 | 0.484 |
| **Relative Δ ApoB, %** | -3.7 (-6.0 to -1.2) | -3.6 (-6.1 to -1.5) | -4.1 (-6.0 to -1.1) | -3.7 (-5.4 to 0.0) | -3.7 (-6.1 to -1.5) | 0.983 | 0.916 |
| **Absolute Δ ApoB/ApoAI** | -0.01 (-0.02 to 0.00) | -0.01 (-0.02 to 0.00) | -0.01 (-0.02 to 0.00) | -0.01 (-0.02 to 0.01) | -0.01 (-0.02 to 0.00) | 0.673 | 0.457 |
| **Relative Δ ApoB/ApoAI, %** | -1.7 (-3.6 to 0.0) | -1.5 (-3.3 to 0.0) | -1.8 (-3.6 to 0.0) | -1.5 (-3.2 to 1.1) | -1.9 (-4.0 to 0.0) | 0.407 | 0.169 |
| Data are absolute frequency (proportion in %) and median (first quartile–third quartile).  **Legend:** Q – Quartile; TC – Total cholesterol; HDL-C – High-density lipoprotein cholesterol; LDL-C – Low-density lipoprotein cholesterol; TAG – Triglycerides; ApoAI – Apolipoprotein AI; ApoB – Apolipoprotein B; Δ – change between 0 min and 120 min (negative value means a reduction of the parameter during test).  **Footnotes:** *Q1 represents individuals with the highest insulin resistance; † p-value for comparisons between all four groups; § p-value for comparisons between Q1 and Q4; ^a^ – Kruskal-Wallis Test; ^b^ – Mann-Whitney Test; ^c^ – Independent Samples T-test.  Post-hoc comparisons for significant results were performed using Dunn's test for Kruskal-Wallis, with p-values adjusted using the Benjamini-Hochberg method (significance set at adjusted p < 0.05). Detailed results for significant comparisons are provided below (parameter name: adjusted p-value for Q2 vs. Q1, Q3 vs. Q1, Q4 vs. Q1, Q3 vs. Q2, Q4 vs. Q2, Q4 vs. Q3): HDL-C at 0 min: 0.153, 0.010, <0.001, 0.231, 0.037, 0.296; TAG at 0 min: 0.014, <0.001, <0.001, 0.029, <0.001, <0.001; HDL-C at 120 min: 0.038, 0.002, <0.001, 0.264, 0.038, 0.264; TAG at 120 min: 0.006, <0.001, <0.001, 0.053, <0.001, <0.001; Relative Δ TAG: 0.145, 0.058, <0.001, 0.522, 0.052, 0.125; ApoB at 0 min: 0.113, 0.002, <0.001, 0.113, 0.032, 0.464; ApoB/ApoAI at 0 min: 0.037, <0.001, <0.001, 0.125, 0.085, 0.782; ApoB at 120 min: 0.099, 0.005, <0.001, 0.229, 0.025, 0.237; ApoB/ApoAI at 120 min: 0.013, <0.001, <0.001, 0.227, 0.070, 0.502. | | | | | | | |

### Correlation between lipid homeostasis parameters and WBIR


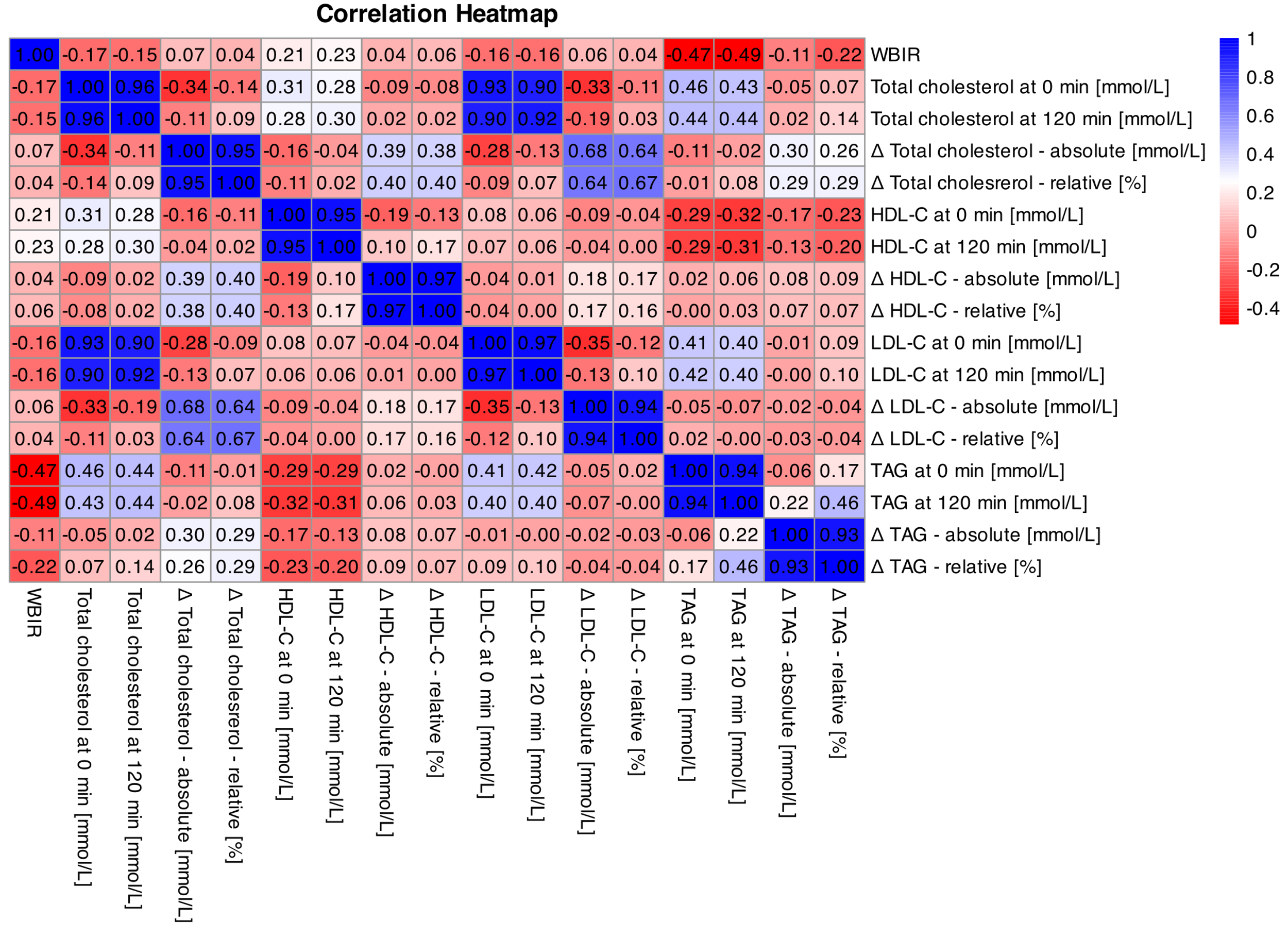


Supplementary Figure 2: Correlation Heatmap for WBIR, Total cholesterol, HDL-C, LDL-C, and TAG.

## Low-grade inflammation


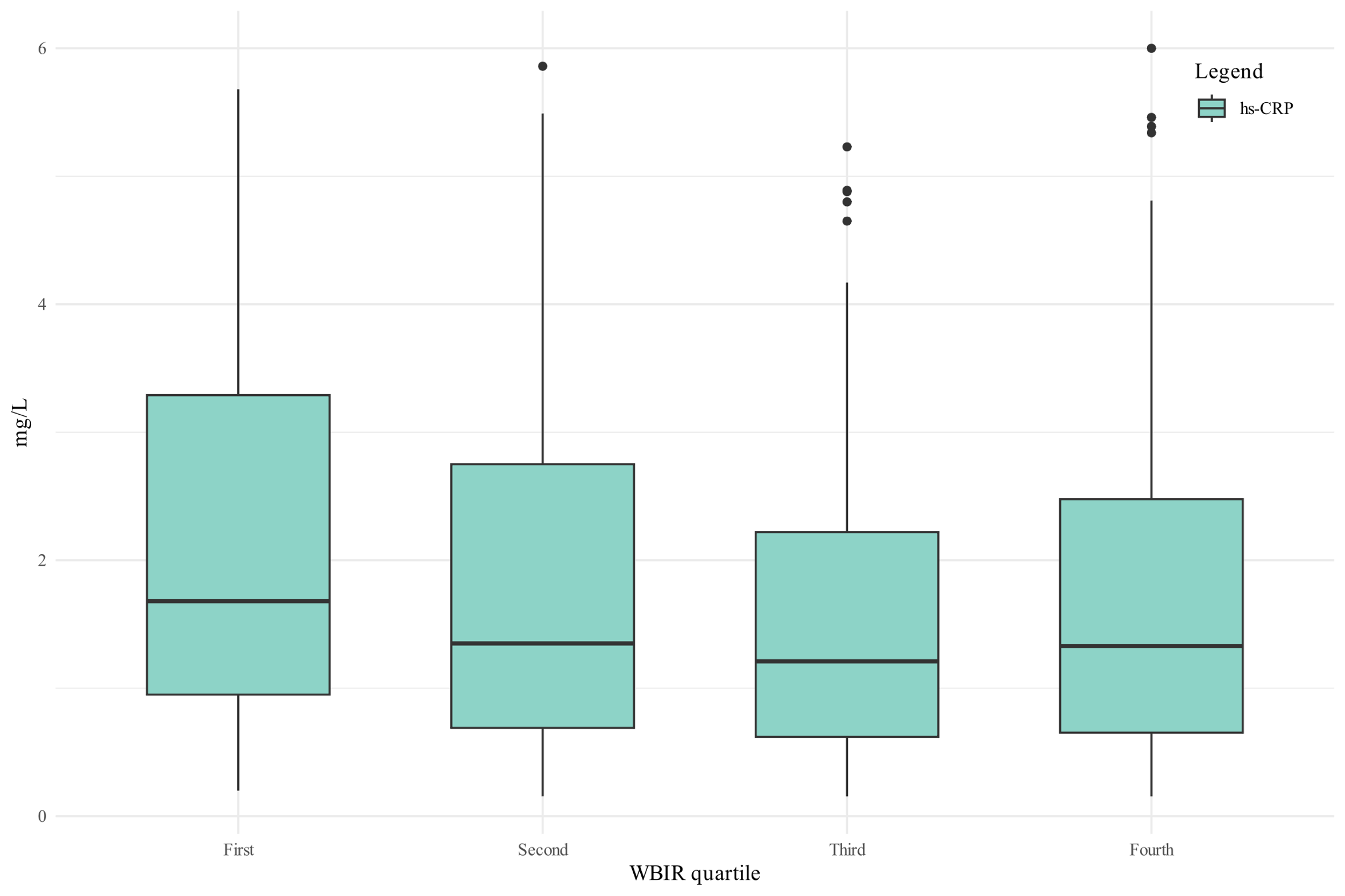


Supplementary Figure 3: High sensitivity C-reactive protein (hs-CRP) as a marker of low-grade inflammation and whole-body insulin resistance (WBIR). Note: Participants in the first WBIR quartile have the highest IR.

Supplementary Table 5: Low-grade inflammation.

|  | **Overall** | **Q1** | **Q2** | **Q3** | **Q4** | **p-value**†**^a^** | **p-value**§**^b^** |
| --- | --- | --- | --- | --- | --- | --- | --- |
| **hs-CRP, mg/L** | 1.38 [0.72–2.74] | 1.68 [0.94–3.36] | 1.35 [0.69–2.75] | 1.21 [0.62–2.26] | 1.33 [0.64–2.57] | 0.043 | 0.057 |
| Data are absolute frequency (proportion in %) and median (first quartile–third quartile).  **Legend:** Q – Quartile; hs-CRP – High sensitivity C-reactive protein.  **Footnotes:** † p-value for comparisons between all four groups; § p-value for comparisons between Q1 and Q4; ^a^ - Kruskal-Wallis Test; ^b^ - Mann-Whitney Test  Post-hoc comparisons for significant results were performed using Dunn's test for Kruskal-Wallis, with p-values adjusted using the Benjamini-Hochberg method (significance set at adjusted p < 0.05). Detailed results for significant comparisons are provided below (parameter name: adjusted p-value for Q2 vs. Q1, Q3 vs. Q1, Q4 vs. Q1, Q3 vs. Q2, Q4 vs. Q2, Q4 vs. Q3): hs-CRP: 0.070, 0.020, 0.070, 0.485, 0.990, 0.485. | | | | | | | |

# Established insulin resistance markers

Supplementary Table 6: Established insulin resistance markers

|  | **Overall** | **Q1*** | **Q2** | **Q3** | **Q4** | **p-value**†**^a^** | **p-value**§**^b^** |
| --- | --- | --- | --- | --- | --- | --- | --- |
| **TAG at 120 min, mmol/L** | 1.00 (0.70–1.40) | 1.30 (1.00–1.70) | 1.10 (0.80–1.40) | 0.90 (0.70–1.28) | 0.70 (0.50–1.00) | <0.001 | <0.001 |
| **AST/ALT ratio** | 0.95 (0.74–1.22) | 0.80 (0.61–0.97) | 0.92 (0.73–1.13) | 0.96 (0.75–1.25) | 1.17 (0.96–1.32) | <0.001 | <0.001 |
| **GGT, μkat/L** | 0.27 (0.20–0.35) | 0.33 (0.25–0.53) | 0.29 (0.23–0.36) | 0.25 (0.18–0.31) | 0.22 (0.17–0.27) | <0.001 | <0.001 |
| **TAG-to-HDL-C ratio at 0 min** | 1.00 (0.64–1.30) | 1.30 (0.91–1.80) | 1.08 (0.70–1.45) | 1.00 (0.64–1.18) | 0.64 (0.47–1.00) | <0.001 | <0.001 |
| **ApoB-to-LDL-C ratio at 0 min** | 0.30 (0.28–0.32) | 0.31 (0.29–0.33) | 0.30 (0.29–0.33) | 0.30 (0.28–0.32) | 0.29 (0.28–0.31) | <0.001 | <0.001 |
| Data are absolute frequency (proportion in %) and median (first quartile–third quartile).  **Legend:** Q – Quartile; SPISE–Single Point Insulin Sensitivity Estimator; TAG – Triglycerides; AST/ALT ratio–Aspartate Aminotransferase to Alanine Aminotransferase ratio; GGT–gamma-glutamyl transpeptidase; TAG-to-HDL-C ratio–Triglycerides to High-Density Lipoprotein Cholesterol ratio; ApoB-to-LDL-C ratio–Apolipoprotein B to Low-Density Lipoprotein Cholesterol ratio.  **Footnotes:** *Q1 represents individuals with the highest insulin resistance; † p-value for comparisons between all four groups; § p-value for comparisons between Q1 and Q4; ^a^–Kruskal-Wallis Test; ^b^–Mann-Whitney Test.  Post-hoc comparisons for significant results were performed using Dunn's test for Kruskal-Wallis, with p-values adjusted using the Benjamini-Hochberg method (significance set at adjusted p < 0.05). Detailed results for significant comparisons are provided below (parameter name: adjusted p-value for Q2 vs. Q1, Q3 vs. Q1, Q4 vs. Q1, Q3 vs. Q2, Q4 vs. Q2, Q4 vs. Q3): TAG at 120 min: 0.006, <0.001, <0.001, 0.053, <0.001, <0.001; AST/ALT ratio: 0.005, <0.001, <0.001, 0.341, <0.001, <0.001; GGT: 0.016, <0.001, <0.001, 0.015, <0.001, 0.036; TAG-to-HDL-C ratio at 0 min: 0.005, <0.001, <0.001, 0.047, <0.001, <0.001; ApoB-to-LDL-C ratio at 0 min: 0.156, 0.012, <0.001, 0.217, 0.015, 0.181. | | | | | | | |


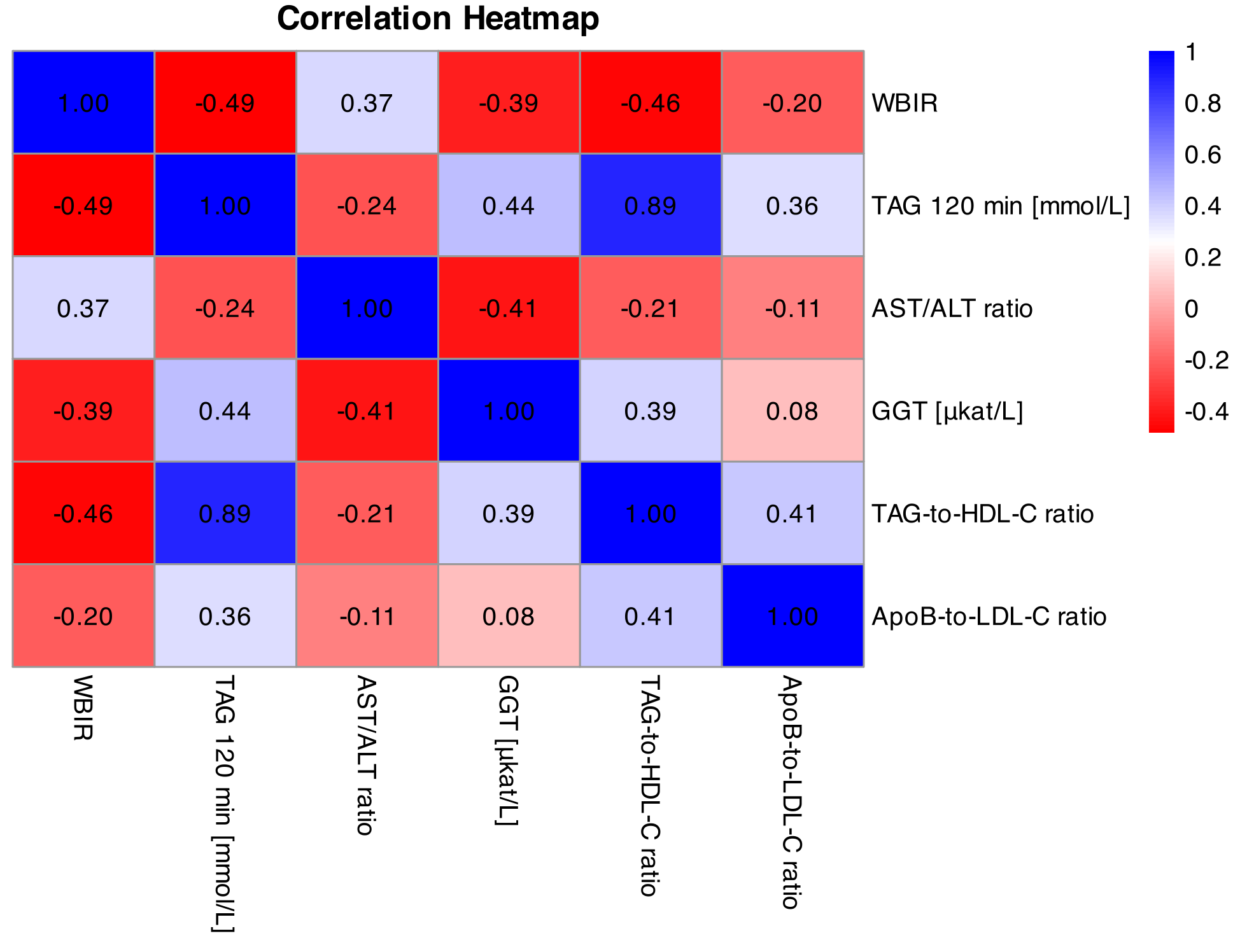


Supplementary Figure 4: Correlation heatmap of established biomarkers of insulin resistance and whole body insulin resistance. Note: the numbers in the rectangles represent the Spearman's rank correlation coefficients.

# REFERENCES

1. Marshall WA, Tanner JM. Variations in pattern of pubertal changes in girls. *Arch Dis Child*. Jun 1969;44(235):291-303. doi:10.1136/adc.44.235.291

2. Marshall WA, Tanner JM. Variations in the pattern of pubertal changes in boys. *Arch Dis Child*. Feb 1970;45(239):13-23. doi:10.1136/adc.45.239.13

3. Matthews DR, Hosker JP, Rudenski AS, Naylor BA, Treacher DF, Turner RC. Homeostasis model assessment: insulin resistance and beta-cell function from fasting plasma glucose and insulin concentrations in man. *Diabetologia*. Jul 1985;28(7):412-9. doi:10.1007/BF00280883

4. DeFronzo RA, Matsuda M. Reduced time points to calculate the composite index. *Diabetes Care*. Jul 2010;33(7):e93. doi:10.2337/dc10-0646

5. Matsuda M, DeFronzo RA. Insulin sensitivity indices obtained from oral glucose tolerance testing: comparison with the euglycemic insulin clamp. *Diabetes Care*. Sep 1999;22(9):1462-70. doi:10.2337/diacare.22.9.1462

6. Barchetta I, Dule S, Bertoccini L, et al. The single-point insulin sensitivity estimator (SPISE) index is a strong predictor of abnormal glucose metabolism in overweight/obese children: a long-term follow-up study. *J Endocrinol Invest*. Jan 2022;45(1):43-51. doi:10.1007/s40618-021-01612-6

7. Paulmichl K, Hatunic M, Hojlund K, et al. Modification and Validation of the Triglyceride-to-HDL Cholesterol Ratio as a Surrogate of Insulin Sensitivity in White Juveniles and Adults without Diabetes Mellitus: The Single Point Insulin Sensitivity Estimator (SPISE). *Clin Chem*. Sep 2016;62(9):1211-9. doi:10.1373/clinchem.2016.257436

8. Katz A, Nambi SS, Mather K, et al. Quantitative insulin sensitivity check index: a simple, accurate method for assessing insulin sensitivity in humans. *J Clin Endocrinol Metab*. Jul 2000;85(7):2402-10. doi:10.1210/jcem.85.7.6661
